# Ancestry-driven metabolite variation provides insights into disease states in admixed populations

**DOI:** 10.1186/s13073-023-01209-z

**Published:** 2023-07-17

**Authors:** Kaylia M. Reynolds, Andrea R. V. R. Horimoto, Bridget M. Lin, Ying Zhang, Nuzulul Kurniansyah, Bing Yu, Eric Boerwinkle, Qibin Qi, Robert Kaplan, Martha Daviglus, Lifang Hou, Laura Y. Zhou, Jianwen Cai, Saame Raza Shaikh, Tamar Sofer, Sharon R. Browning, Nora Franceschini

**Affiliations:** 1 Department of Biostatistics, University of North Carolina, Chapel Hill, NC USA; 2 Department of Epidemiology, University of North Carolina, 123 W Franklin St, Suite 401, NC NC 27516 Chapel Hill, USA; 3 Department of Biostatistics, University of Washington, Seattle, WA USA; 4 Division of Sleep and Circadian Disorders, Brigham and Women’s Hospital, Boston, MA USA; 5 Human Genetics Center, University of Texas Health Science Center at Houston, Houston, TX USA; 6 Department of Epidemiology and Population Health, Albert Einstein College of Medicine, Bronx, NY USA; 7 Public Health Sciences Division, Fred Hutchinson Cancer Research Center, Seattle, WA USA; 8 Institute for Minority Health Research, University of Illinois at Chicago, Chicago, IL USA; 9 Department of Preventive Medicine, Northwestern University Feinberg School of Medicine, Chicago, IL USA; 10 Department of Nutrition, University of North Carolina, Chapel Hill, NC USA; 11 Departments of Medicine and Biostatistics, Harvard University, Boston, MA USA

**Keywords:** Admixture mapping, Local ancestry, Metabolites, Hispanics/Latino populations

## Abstract

**Background:**

Metabolic pathways are related to physiological functions and disease states and are influenced by genetic variation and environmental factors. Hispanics/Latino individuals have ancestry-derived genomic regions (local ancestry) from their recent admixture that have been less characterized for associations with metabolite abundance and disease risk.

**Methods:**

We performed admixture mapping of 640 circulating metabolites in 3887 Hispanic/Latino individuals from the Hispanic Community Health Study/Study of Latinos (HCHS/SOL). Metabolites were quantified in fasting serum through non-targeted mass spectrometry (MS) analysis using ultra-performance liquid chromatography-MS/MS. Replication was performed in 1856 nonoverlapping HCHS/SOL participants with metabolomic data.

**Results:**

By leveraging local ancestry, this study identified significant ancestry-enriched associations for 78 circulating metabolites at 484 independent regions, including 116 novel metabolite-genomic region associations that replicated in an independent sample. Among the main findings, we identified Native American enriched genomic regions at chromosomes 11 and 15, mapping to *FADS1/FADS2* and *LIPC*, respectively, associated with reduced long-chain polyunsaturated fatty acid metabolites implicated in metabolic and inflammatory pathways. An African-derived genomic region at chromosome 2 was associated with N-acetylated amino acid metabolites. This region, mapped to *ALMS1*, is associated with chronic kidney disease, a disease that disproportionately burdens individuals of African descent.

**Conclusions:**

Our findings provide important insights into differences in metabolite quantities related to ancestry in admixed populations including metabolites related to regulation of lipid polyunsaturated fatty acids and N-acetylated amino acids, which may have implications for common diseases in populations.

**Supplementary Information:**

The online version contains supplementary material available at 10.1186/s13073-023-01209-z.

## Background

Circulating metabolites can provide insights into biological processes in health and disease states [1]. Metabolites are end-products of metabolic cellular pathways regulated by genetic variation and environmental factors such as diet, microbiome, and exposure to exogenous compounds [2]. Studies have identified metabolite profiles for complex diseases including diabetes, obesity, and chronic kidney disease [3, 4]. The integration of genotypes and metabolites has provided insights into disease mechanisms [5]. This research has been facilitated by the generation of high-throughput metabolomics in large datasets with genome-wide genotypes. Using genome-wide association approaches, several large-scale studies have identified rare and common genetic variants regulating blood metabolite levels in populations [6–8], and genes related to disease processes or drug targets [9].

However, the role of genetic ancestry in metabolite abundance and regulation is not well known. This question is relevant to populations with recent admixture, such as Hispanic/Latino populations, who have a high burden of metabolic diseases. Hispanic/Latino populations were shaped by a long history of colonization and migration across America, which introduced high diversity in both cultural aspects and genetic ancestry that can be observed in the Hispanic/Latino individuals currently in the USA [10, 11]. They carry genetic variants from three continental ancestries (West African, European, and Native American), and their genome is composed of varying segments of these different ancestral origins that can be mapped to local ancestries, i.e., each chromosomal segment can be attributed to a source, local (segment-specific) ancestry. Genomic regions associated with a specific ancestry are expected to be enriched for ancestry-derived variants. There are several examples of ancestry-derived genetic variations that confer disease risk in populations, some of them driven by adaptation to environmental factors such as dietary restrictions or exposure to pathogens [12, 13]. For example, in Hispanic/Latino individuals, Native American single nucleotide variants (SNVs) at the *SLC16A11* gene are associated with risk of diabetes, and African ancestry SNVs in the *APOL1* gene (related to plasmodium pathogen resistance) are associated with chronic kidney disease [14, 15]. Therefore, research focusing on genetic ancestry could point to differences in metabolic regulation across populations and provide insight into differences in disease risk.

We leveraged local ancestries for a comprehensive study of ancestry-derived genomic regions associated with circulating metabolites in admixed Hispanic/Latino individuals. Recent admixture creates long-range blocks of linkage disequilibrium (admixture-LD) between genetic variants with differences in allele frequencies in the parental populations. The extent of admixture depends on the population admixture dynamics and time since admixture, and recombination rates. Causal variants should occur more frequently on chromosomal segments inherited from ancestral populations with higher disease burden. Therefore, associations of phenotypes with local ancestry regions are likely to improve the discovery of causal variants enriched in their population of origin for both rare and common SNVs. We studied the genetic ancestry influences on 640 circulating metabolites in 3887 Hispanic/Latino individuals from the Hispanic Community Health Study/Study of Latinos (HCHS/SOL) using admixture mapping. This approach has been successfully applied in HCHS/SOL, with the identification of novel loci associated with blood pressure [16], chronic kidney disease traits [17, 18], and pulmonary traits [19, 20]. We used summary statistics from a published genome-wide association study (GWAS) of metabolites in the HCHS/SOL for fine-mapping loci and replicated novel findings in a non-overlapping HCHS/SOL sample.

## Methods

### HCHS/SOL study design, population, and covariates

The HCHS/SOL is a multi-center community-based prospective cohort study of 16,415 self-identified Hispanic/Latino individuals aged 18–74 years who were recruited from households in predefined census-block groups in four US field centers (Chicago, Miami, the Bronx, and San Diego) between 2008 and 2011 [21]. The study used a two-stage area probability sample of households selected with stratification and oversampling incorporated at each stage to provide a broadly diverse sample of Hispanics/Latinos and to ensure that the target age distribution was obtained [22]. Participants self-reported their country of origin as Central America (*n* = 1730), Cuba (*n* = 2348), Dominican Republic (*n* = 1460), Mexico (*n* = 6471), Puerto Rico (*n* = 2728), or South America (*n* = 1068). A baseline clinical examination included clinical, behavioral, and sociodemographic assessments, and collection of fasting blood and spot urine samples. Estimated glomerular filtration rate (eGFR) was calculated using the race-free Chronic Kidney Disease Epidemiology Collaboration serum creatinine-based equation and used as a covariate in analyses.

### Metabolomics data and processing

We used two non-overlapping datasets sampled from the overall HCHS/SOL study for discovery and replication. The discovery included a sample of 3972 participants which was randomly selected from HCHS/SOL for serum metabolomic profiling. Serum metabolites (*n* = 1136; 782 with known and 354 unknown biochemical identities) were quantified in fasting serum through non-targeted mass spectrometry (MS) analysis using ultra-performance liquid chromatography (UPLC)-MS/MS (DiscoveryHD4™ platform, Metabolon Inc, NC) [23]. Identification and classification of metabolites used a comparison of the ion features in the experimental samples to a reference library of chemical standard entries (e.g., molecular weight (m/z), preferred adducts, in-source fragments, and associated MS spectra) and known chemical entities. Peaks were quantified using area-under-the-curve. Raw area counts for each metabolite in each sample were normalized to correct for variation resulting from instrument inter-day tuning differences. To avoid batch effects, samples were randomly allocated across the platform. Replicas were used to determine endogenous variability, with representative relative standard deviation of 10% across all biochemicals. Metabolites were inverse normally transformed to approximate a normal distribution. We excluded unknown metabolites and metabolites with 25% or more missing data (amino acids, *n* = 12; carbohydrates, *n* = 2; cofactor a, *n* = 5; lipid, *n* = 23; nucleotide, *n* = 2; peptide, *n* = 3; xenobiotics, *n* = 95). For metabolites with less than 25% missing data, missing values were imputed with the observed minimum value of the metabolite in the sample. A total of 640 metabolites were used in the analyses. We provided the Human Metabolome Database (HMDB) annotation when available and used LIPID MAPS to provide a RefMet-driven lipid standardized name [24].

The replication dataset included 2,330 HCHS/SOL participants who were profiled using the DiscoveryHD4™ platform (Metabolon Inc, NC) using the same protocols described for the discovery dataset. After removing individuals overlapping with the discovery dataset, duplicates, and individuals without genotypes, 1856 nonoverlapping participants with the discovery sample were used for replication. We excluded metabolites with > 25% missing values across individuals and imputed missing values for the remaining metabolites using the same methods as described in the discovery dataset.

### Genotyping and local ancestry

Participants were genotyped at over 2.5 million SNVs using a custom-built Illumina array. Details on genotyping, quality control proceedings, and imputation have been published [25–27]. Local ancestry references were estimated from 195 West African, 527 European, and 63 Native American samples from the Human Genome Diversity Project [28] and 1000 Genomes Project [29]. BEAGLE (v.4) was employed for phasing and imputation of sporadic missing genotypes in the HCHS/SOL and reference-panel datasets [30]. Local ancestry calls at each locus were estimated using RFMix 1.5.4 [31], with the PopPhased option and a minimum node size of 5, as recommended in the documentation, and previously described [27]. The estimation of kinship coefficients, principal components (PCs) of ancestry, and genetic analysis groups are published [25].

### Statistical analyses

Descriptive statistics for continuous data were presented as the mean ± standard deviation (SD) or median with interquartile range, and categorical variables as number and percentage. The HCHS/SOL study was developed under a complex sampling design [22]. To account for the correlation structure of the data, all regression models included three random effects: the pairwise kinship coefficients, household, and census block group (the geographic cluster of the households) to represent the correlation between participants to genetic relatedness and shared environmental effects.

### Admixture mapping

Admixture mapping analyses for each metabolite were performed using a linear mixed model framework implemented in the GENESIS R package [32] in which African, European, and Native American ancestries were tested simultaneously in a joint admixture mapping analysis [18]. Briefly, we first fit the models under the null hypothesis of no genetic ancestry effect while including multiple random (pairwise kinship coefficients, household, and census block group) and fixed (age, sex, eGFR, recruitment center, genetic analysis group, and the first five PCs) effects. We then used the null models to run the joint test for associations between ancestries at each locus and metabolites using a Wald test. Here, local ancestry is defined as the locus-specific ancestry allelic dosages (0, 1, or 2 copies of African, European, or Native American alleles, estimated from the genotype data) at each genomic interval. After identifying associations using the joint test, follow-up admixture mapping analyses then tested each ancestry against the others to determine the ancestry driving the signal at each associated local ancestry region. We tested a total of 15,500 local ancestry regions. We applied a significance threshold of 5.04 × 10^−9^, which controls for a family-wise error rate at a level of 0.05 for multiple testing for the 640 metabolites and 15,500 local ancestry regions. The effect sizes of the associated loci were estimated using the allelic dosage of the ancestry driving the signal.

In a sensitivity analysis, we ran the admixture mapping models adjusting for global ancestry, defined as ancestry proportions estimated by averaging the local ancestry calls across the genome, instead of using the first five PCs, to ensure that our significant local ancestry associations were not spurious. We obtained similar results adjusting by either PC or global ancestry proportions, and although 18 local ancestry regions were no longer significant, *p*-values had small changes. The sensitivity analysis results indicated that the PCs accurately adjusted for population structure and increased our confidence that observed associations were not spurious.

### Conditional analysis of admixture mapping regions

RFMix infers local ancestry in a series of intervals, referred to here as local ancestry regions. Inferred local ancestry is constant within each region. However, local ancestry is not independent across neighboring regions because local ancestry tracts have average lengths of more than six centiMorgans in Hispanic individuals due to the onset of admixture occurring within the past 15 generations. To determine whether significant results from local ancestry regions located close in proximity were independent, we performed a conditional admixture mapping analysis on local ancestry regions located within ten centiMorgans of the most significant region associated with a metabolite. The allelic dosage of the ancestry driving the association signal was included as a covariate. We conservatively chose a significance threshold of 5 × 10^−5^ for this analysis and kept only ancestry-derived regions that remained significantly associated with a metabolite after conditioning.

### Overlap of local ancestry regions with genetic variants identified in GWAS

We used the recently published GWAS of metabolites to identify the overlap of significant local ancestry regions with genetic variants significant in GWAS [33]. Local ancestry regions without any significant GWAS variants were considered novel associations discovered through admixture mapping.

### Conditional analyses using GWAS summary statistics of metabolites in HCHS/SOL for fine-mapping

We used existing GWAS summary statistics from the HCHS/SOL cohort to fine-map potential variants within identified ancestry-specific regions associated with metabolites. The methods for the GWAS were described previously [8]. We extracted GWAS results for all SNVs located within significant local ancestry regions. We then used the conditional and joint association analysis (COJO) application from the software package Genome-wide Complex Trait Analysis (GCTA v1.93.2) to determine which SNVs (separately for each local ancestry region) were independent. The number of copies of the reference allele for each independent SNV was included as a covariate in a conditional admixture mapping analysis for its corresponding region [34]. The reference population for calculating linkage disequilibrium was a sample of 5879 unrelated individuals from the HCHS/SOL study. Only GWAS SNVs from COJO modeling with p-values lower than 5 × 10^−8^ were included as covariates in admixture mapping conditional analyses. We considered a SNV to explain the admixture mapping association with a metabolite if the admixture mapping joint p-value increased to greater than 5 × 10^−5^ when conditioning on the SNV. For local ancestry regions with multiple COJO SNVs, if none of the COJO variants individually changed the admixture mapping joint p-value to more than 5 × 10^−5^, all of the COJO SNVs for that region were tested together in a single admixture mapping conditional model.

### Genotype annotation

We used several tools to annotate SNVs identified in conditional analysis for their functional impact including ANNOVAR [35] and the Ensembl (http://uswest.ensembl.org/index.html) and refGene annotation databases (https://varianttools.sourceforge.net/Annotation/RefGene). For non-coding variants, we used resources to assess evidence for enrichment in regulatory elements, including enhancers, transcription factor binding sites, and histone modification in tissues using a range of approaches implemented in FORGE2 [36].

### Replication of admixture mapping findings

We performed admixture mapping in an independent sample from HCHS/SOL using the same statistical methods and covariates used in the discovery analysis. The threshold for significance was based on Bonferroni correction for 404 association tests of 64 metabolites with associations spread across 169 genomic regions. In addition, we compared the direction of effects (beta coefficients) and the ancestry driving the association between the discovery and replication samples.

## Results

### Admixture mapping of metabolites

We performed admixture mapping of metabolites in 3887 HCHS/SOL Hispanic/Latino individuals. The mean age was 45.9 years old, 57% were women, and the mean eGFR was 96.4 ml/min/1.73 m^2^ (Additional File 1: Table S1). Participants lived in the USA and reported originated from the Mainland (Central and South America, and Mexico) and the Caribbean (Cuba, Dominican Republic, and Puerto Rico), and they had varying African, European, and Native American global ancestry proportions, as shown in Additional File 2: Fig. S1 and previously described [25]. Note that individuals from the Mainland had a higher proportion of Native American ancestry, while those from the Caribbean had a higher proportion of African ancestry.

**Fig. 1:**
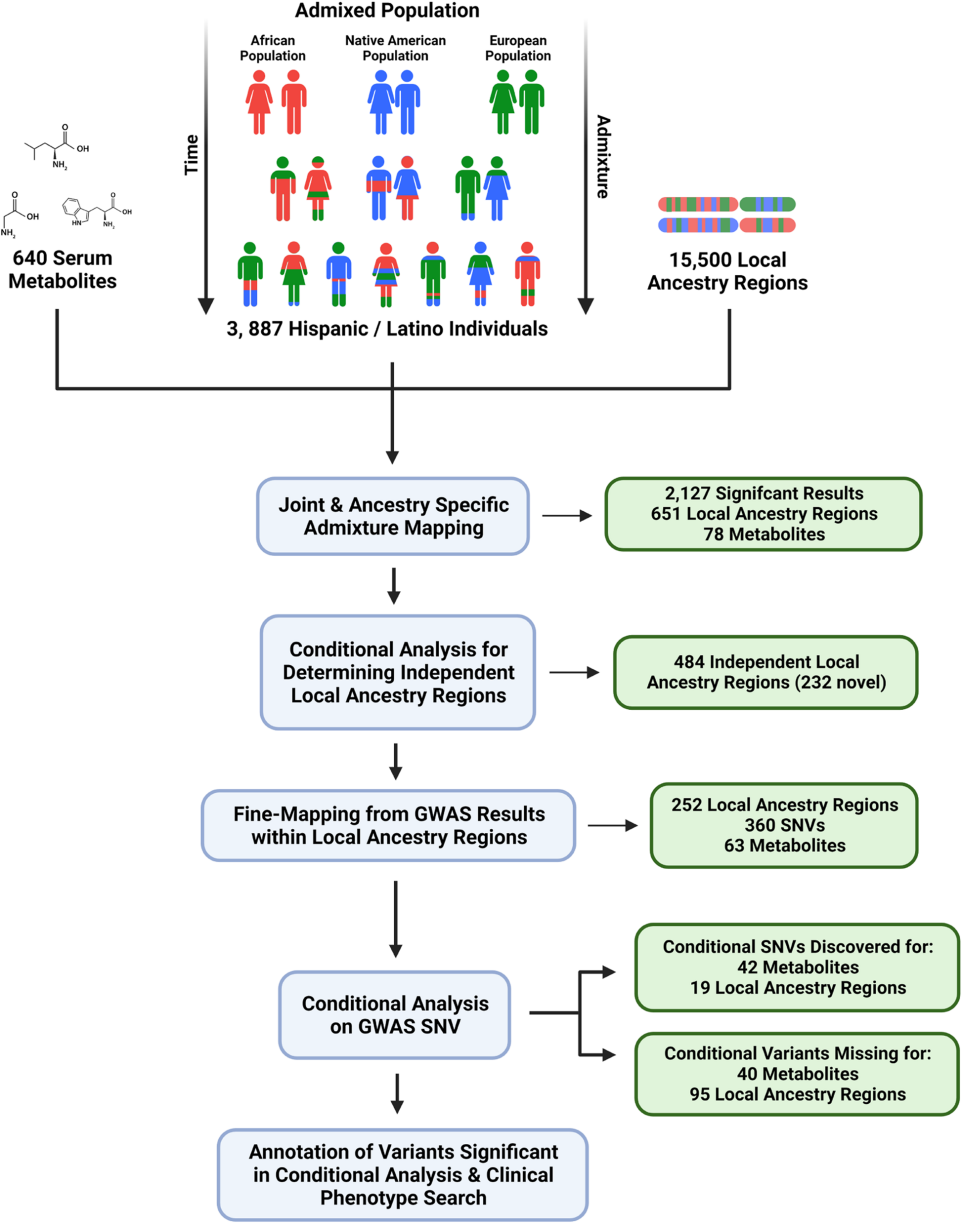
Study design and results. Hispanic/Latino populations are descendants from African, European, and Native American ancestral populations. Joint (all-ancestry) and ancestry-specific admixture mapping was performed using genetic, metabolic, and clinical data from 3887 Hispanic/Latino individuals. Conditional admixture mapping analysis determined individual local ancestry regions. These local ancestry regions were fine-mapped using GWAS results from a previous study. Annotation and clinical phenotypes were obtained for significant variants from conditional admixture mapping analysis

The overall study design and approach for analyses are shown in Fig. 1. Associations were performed among 640 metabolites with known biochemical identities and 15,500 local ancestry regions using a joint admixture mapping linear mixed model. A total of 651 local ancestry regions within twelve chromosomes were significantly associated with at least one metabolite, and 78 of the 640 tested metabolites were associated with at least one significant local ancestry region (Additional File 1: Table S2). Summarizing associations while allowing for multiple metabolite associations per region, our study identified a total of 2127 significant metabolite-local ancestry pair results (Additional File 1: Table S3).

### Local ancestry independent associations

Given that several metabolites were frequently associated with multiple neighboring local ancestry regions, we tested associations conditioning on local ancestry regions of the most significant region, which reduced the number of significant local ancestry regions from 2127 to 484 independent regions (Additional File 3: Extended Data Table S1). The remaining local ancestry regions were removed from future analyses.

### Ancestry-driven regions for metabolites

**Fig. 2:**
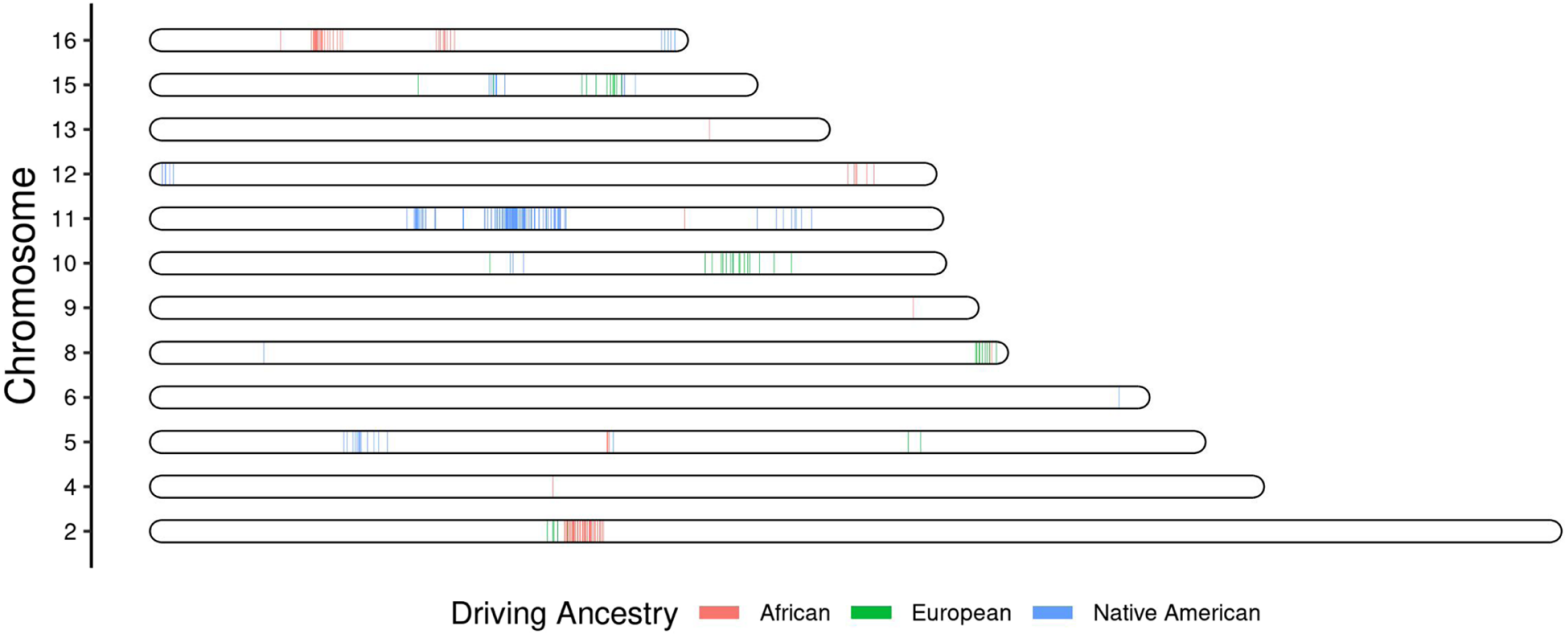
Location of associated independent local ancestry regions. Each vertical line represents an independent local ancestry region, colored by driving ancestry (the ancestry with the lowest *p*-value in ancestry-specific analyses). Regions located in relatively close proximity tend to have the same driving ancestry

We next tested the ancestry driving the association in each independent local ancestry region. The most significant ancestry associations were attributed to Native American ancestry (Table 1). There was notable clustering of association regions by ancestry where multiple nearby independent regions across metabolites were attributed to the same ancestry (Fig. 2). On several chromosomes, there was a concordant association between the driving ancestry and the direction of the association, i.e., one ancestry was associated with increased metabolite abundance while the other was associated with reduced metabolite abundance (Additional File 2: Fig. S2). A large number of metabolites were associated with two local ancestry regions located on chromosomes 2 and 11. Of the metabolites, 97.4% of those associated with regions on chromosome 11 were lipids, and 84.6% of those associated with chromosome 2 were amino acids (Table 1). On chromosome 2, 91% of the associations were attributed to African local ancestry, with 89% of those associations being related to increased metabolite abundance. Similarly, on chromosome 11, all but one of the 264 local ancestry region associations were driven by Native American ancestry, with 86% of these associations being related to low metabolite quantities (Table 1).

**Table 1:** All admixture mapping results by ancestry and super pathway in independent local ancestry regions

		**Significant LA regions**		**Driving ancestry from ancestry-specific analysis**			**Super pathway**		
**Chr**	**Metabolites** ***n***	**Novel** ***n* (%)**	**Total** ***n***	**NAM** ***n* (%)**	**AFR** ***n* (%)**	**EUR** ***n* (%)**	**Lipids** ***n* (%)**	**Amino acids** ***n* (%)**	**Other** ***n* (%)**
2	13	41 (48)	85	-	78 (92)	7 (8)	1 (8)	11 (85)	1 (7)
4	1	-	1	-	1 (100)	-	1 (100)	-	-
5	3	14 (74)	19	14 (74)	3 (16)	2 (11)	-	2 (67)	1 (33)
6	1	-	1	1 (100)	-	-	-	-	1 (100)
8	4	8 (53)	15	1 (7)	5 (33)	9 (60)	-	4 (100)	-
9	1	-	1	-	1 (100)	-	-	-	1 (100)
10	2	13 (65)	20	3 (15)	-	17 (85)	1 (50)	-	1 (50)
11	38	114 (70)	164	263 (99.6)	1 (0.4)	-	37 (97)	1 (3)	-
12	4	7 (58)	12	6 (50)	6 (50)	-	2 (50)	2 (50)	-
13	1	-	1	-	1 (100)	-	-	-	1 (100)
15	8	15 (58)	26	14 (54)	-	12 (46)	6 (75)	2 (25)	-
16	4	20 (51)	39	6 (15)	33 (85)	-	-	3 (75)	1 (25)

Novel regions are local ancestry regions containing no significant SNVs from GWAS. The total number of significant local ancestry regions allows for duplicate significant regions across metabolites for the corresponding chromosome. Ancestry-specific analysis was performed on all metabolites significant in joint (all-ancestries) admixture mapping. The driving ancestry was the ancestry group with the smallest *p*-value in ancestry-specific testing. Metabolic super pathways represent overall metabolite function in the body

*Chr* chromosome, *LA* local ancestry, *NAM* Native American driving ancestry, *AFR* African driving ancestry, *EUR* European driving ancestry

### Fine-mapping results using GWAS variants on genomic regions overlapping with GWAS findings

Among 484 metabolite-genomic associations, 252 (51%) of the local ancestry regions overlapped with a significant variant reported in GWAS, and 232 without overlap were considered novel. We used GWAS summary statistics for metabolites in HCHS/SOL from a recent publication [8] to test if genome-wide significant SNVs explained our admixture mapping findings. Using a stepwise selection model implemented in GCTA-COJO, we first identified all independent associated SNVs within the boundaries of each local ancestry region. These analyses identified 361 SNVs in the 252 local ancestry regions, which were then used in admixture mapping conditional analyses.

**Fig. 3:**
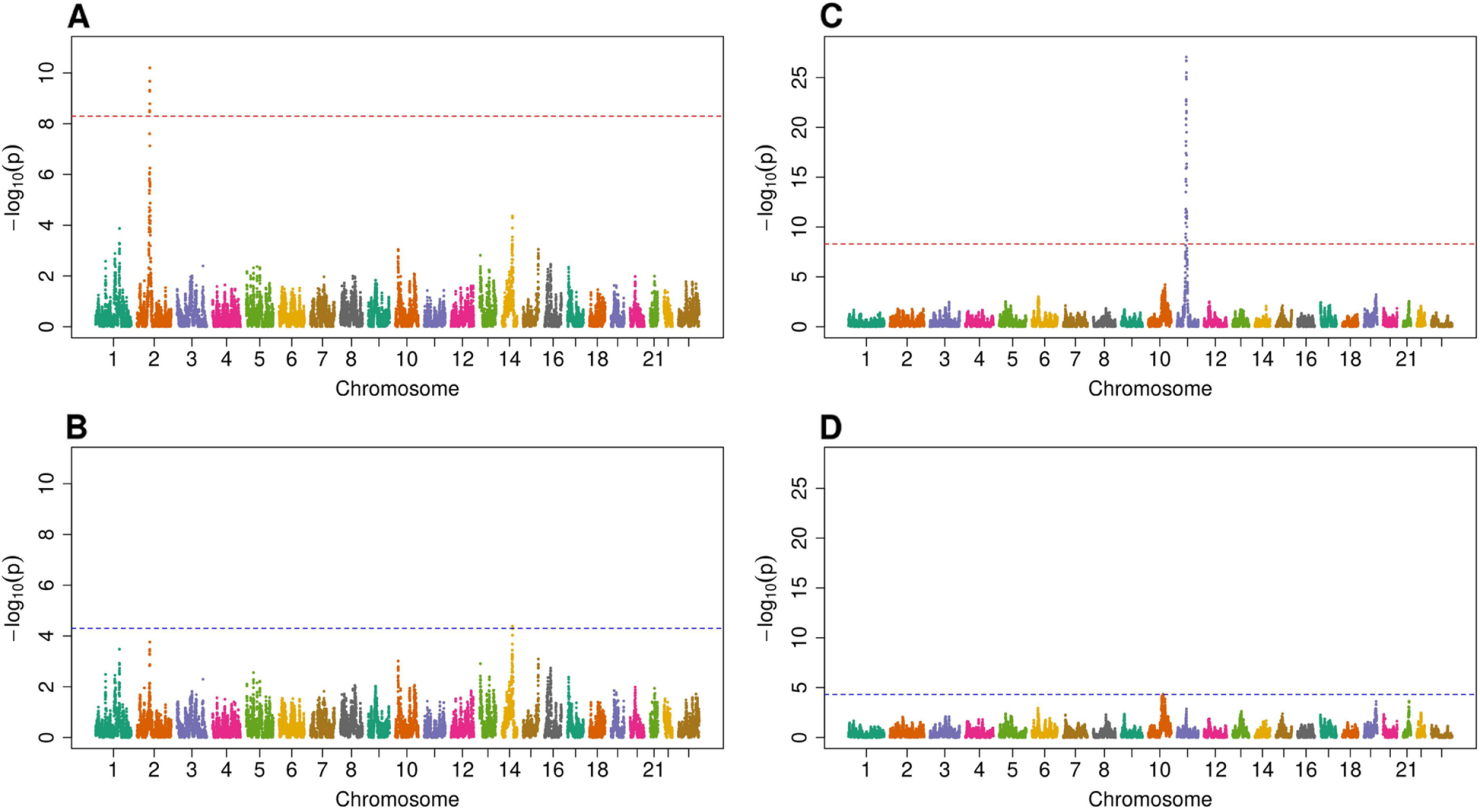
COJO SNVs account for admixture mapping signals. The horizontal red line in **A** and **C** represents the significance level of 5.04 × 10^−9^ for all-ancestry admixture mapping. The horizontal blue line in **B** and **D** represents the significance level of 5 × 10^−5^ for conditional admixture mapping. **A** Admixture mapping results for 2-aminooctanoic acid. **B** The SNV rs72903325 accounts for the admixture mapping signal in local ancestry region 1581 on chromosome 2 for 2-aminooctanoic acid. **C** Admixture mapping results for arachidonoylcholine. **D** The SNV rs174567 accounts for the admixture mapping signal in local ancestry region 9120 on chromosome 11 for arachidonoylcholine

Among these SNVs, 46 explained the admixture association in 42 local ancestry regions (Table 2), and two examples are shown in Fig. 3. The significance of a locus was explained in a further two local ancestry regions when conditioning on all COJO-selected SNVs in a region (Additional File 1: Table S4).

**Table 2:** COJO genetic variants that explain association between local ancestry regions and metabolites

Metabolite	Chr	LA ID	COJO rsID	Gene	Gene function	Driving ancestry (MAF)	AM joint *P*-value	Conditional joint *P*-value
Alliin	2	1579	rs10189885	*ALMS1P1*	Intronic	EUR (—)	2.77 × 10^−10^	9.00 × 10^−2^
N-acetylkynurenine (2)	2	1579	rs10189885^a^	*ALMS1P1*	Intronic	AFR (—)	4.13 × 10^−12^	2.87 × 10^−1^
N2-acetyllysine	2	1579	rs12620091	*ALMS1P1*	Intronic^b^	AFR (0.17)	2.87 × 10^−17^	1.19 × 10^−3^
N-acetylglutamine	2	1579	rs13431529	*ALMS1P1*	Intronic^b^	AFR (0.6)	6.05 × 10^−12^	6.29 × 10^−1^
N2-acetyllysine	2	1579	rs1881244	*ALMS1*	Intronic	AFR (0.85)	2.87 × 10^−17^	3.47 × 10^−3^
N-acetylarginine	2	1579	rs1881244	*ALMS1*	Intronic	AFR (0.85)	4.85 × 10^−25^	3.75 × 10^−3^
N-acetylasparagine	2	1579	rs1881244	*ALMS1*	Intronic	AFR (0.85)	1.74 × 10^−22^	7.78 × 10^−4^
N-acetylglutamine	2	1579	rs1881244	*ALMS1*	Intronic	AFR (0.85)	6.05 × 10^−12^	1.42 × 10^−2^
N-acetylleucine	2	1579	rs1881244	*ALMS1*	Intronic	AFR (0.85)	2.07 × 10^−19^	1.52 × 10^−4^
N-acetyltyrosine	2	1579	rs1881244	*ALMS1*	Intronic	AFR (0.85)	1.73 × 10^−14^	1.04 × 10^−1^
N-delta-acetylornithine	2	1579	rs28525015	*ALMS1P1*	Intronic^b^	AFR (0.61)	6.15 × 10^−24^	3.17 × 10^−4^
N-acetylleucine	2	1579	rs28879089	*ALMS1*	Intronic	AFR (—)	2.07 × 10^−19^	6.41 × 10^−3^
N-acetyltyrosine	2	1579	rs6546854	*ALMS1*	Intronic	AFR (0.62)	1.73 × 10^−14^	1.86 × 10^−1^
2-Aminooctanoic acid	2	1581	rs72903325	*ACTG2*	Intronic	AFR (0.35)	6.26 × 10^−11^	1.73 × 10^−4^
Androstenediol (3alpha, 17alpha) monosulfate (3)	4	3592	rs138040976	*GNRHR*	Intronic	AFR (—)	4.07 × 10^−11^	3.10 × 10^−4^
3-aminoisobutyrate	5	4386	rs191679549	*AGXT2*	Intronic	NAM (—)	7.11 × 10^−29^	6.87 × 10^−3^
Betaine	5	4546	rs7736027	*DMGDH*	Intronic	AFR (0.66)	2.16 × 10^−11^	2.12 × 10^−2^
N-acetylglucosaminylasparagine	6	5848	rs2346120^a^	*UNC93A*	Intronic	NAM (—)	3.20 × 10^−9^	1.67 × 10^−2^
S-1-pyrroline-5-carboxylate	8	7373	rs2242090	*PYCR3*	Exonic^c^	EUR (0.52)	1.43 × 10^−24^	1.01 × 10^−1^
6-oxopiperidine-2-carboxylate	8	7376	rs11993782	*PLEC*	Intronic	AFR (0.83)	2.24 × 10^−10^	9.18 × 10^−2^
2′-O-methyluridine	9	7977	rs56023505	*PHYHD1*	UTR5	AFR (0.44)	3.35 × 10^−10^	4.56 × 10^−3^
Carnitine	10	8412	rs12415764	*ARID5B*	Intergenic	NAM (0.24)	2.32 × 10^−11^	5.37 × 10^−5^
N-methylpipecolate	10	8587	rs2147896	*PYROXD2*	Exonic^c^	EUR (0.35)	2.02 × 10^−46^	1.39 × 10^−3^
MG 20:4	11	9120	rs102274	*TMEM258*	Intronic	NAM (0.59)	2.15 × 10^−37^	5.72 × 10^−3^
PC 16:0/18:2	11	9120	rs11320420	*MYRF*	Intronic	NAM (—)	1.53 × 10^−15^	6.34 × 10^−2^
Stearidonic acid	11	9120	rs174556	*FADS1*	Intronic	NAM (0.57)	1.30 × 10^−19^	3.60 × 10^−4^
1-arachidonoyl-GPC (20:4n6)^b^	11	9120	rs174562	*FADS2*	Intronic	NAM (0.59)	7.02 × 10^−76^	1.02 × 10^−4^
PC 16:0/20:4	11	9120	rs174562	*FADS2*	Intronic	NAM (0.59)	1.04 × 10^−69^	1.72 × 10^−4^
Arachidonoylcholine	11	9120	rs174567	*FADS2*	Intronic	NAM (0.61)	8.82 × 10^−28^	9.13 × 10^−3^
PC 18:2/18:2	11	9120	rs3834458	*FADS2*	Intronic	NAM (0.58)	5.97 × 10^−26^	4.87 × 10^−3^
LPC 18:2/0:0	11	9120	rs3834458	*FADS2*	Intronic	NAM (0.58)	8.16 × 10^−11^	1.23 × 10^−1^
Sphingomyelin (d18:1/20:2, d18:2/20:1, d16:1/22:2)^b^	11	9120	rs3834458	*FADS2*	Intronic	NAM (0.58)	2.24 × 10^−10^	1.61 × 10^−2^
PE 16:0/18:2	11	9120	rs5792235	*FADS2*	Intronic	NAM (0.59)	5.40 × 10^−29^	4.02 × 10^−2^
PC 18:0/18:2	11	9120	rs5792235	*FADS2*	Intronic	NAM (0.59)	3.80 × 10^−11^	1.43 × 10^−1^
PE 18:0/18:2	11	9120	rs5792235	*FADS2*	Intronic	NAM (0.59)	2.32 × 10^−22^	7.67 × 10^−2^
LPC P-16:0 or LPC O-16:1	11	9121	rs11230873	*FTH1*	Intergenic	NAM (0.26)	1.60 × 10^−10^	9.49 × 10^−5^
MG 20:4	11	9149	rs142021492	*—*	—	NAM (—)	2.93 × 10^−9^	2.21 × 10^−3^
3beta-hydroxy-5-cholestenoate	11	9317	chr11:110244360	*RDX*	—	NAM (—)	6.11 × 10^−26^	4.75 × 10^−2^
Ethylmalonate	12	10132	chr12:121176083	*ACADS*	Exonic^c^	AFR (—)	5.72 × 10^−13^	1.41 × 10^−1^
Ethylmalonate	12	10,132	rs73415734	*—*	—	AFR (—)	5.72 × 10^−13^	1.82 × 10^−2^
LPE 16:0/0:0	15	11487	rs1077834	*LIPC*	Intronic^c^	NAM (0.43)	2.32 × 10^−14^	2.90 × 10^−3^
LPE 18:0/0:0	15	11487	rs1077834	*LIPC*	Intronic^c^	NAM (0.43)	1.85 × 10^−11^	4.26 × 10^−2^
PE 16:0/20:4	15	11487	rs2070895	*LIPC*	Intronic	NAM (—)	5.53 × 10^−18^	3.20 × 10^−2^
PE 18:0/20:4	15	11487	rs2070895	*LIPC*	Intronic	NAM (—)	5.10 × 10^−13^	1.98 × 10^−1^
Cys-gly, oxidized	16	12348	chr16:89909429	*SPIRE*	Intronic	NAM (—)	1.46 × 10^−29^	4.68 × 10^−2^
Cysteinylglycine	16	12348	rs62068366	*SPATA2L*	Intronic	NAM (0.28)	8.45 × 10^−10^	3.14 × 10^−2^

*COJO* conditional and joint association analysis, *Chr* chromosome, *LA ID* local ancestry region ID, *MAF* minor allele frequency, *AFR* African, *NAM* Native American, *EUR* European, *AM* admixture mapping

^a^Genetic variant not significant in GWAS

^b^ncRNA

^c^Missense

^c^Upstream

Interestingly, 31 significant local ancestry associations with metabolites were driven by only four local ancestry regions on chromosomes 2, 11, 15, and 16. Often, the same SNV explained the association with local ancestry for multiple metabolites (Additional File 1: Tables S5 to S8). With only one exception (alliin on chromosome 2), local ancestry associations explained by the same SNV in multiple metabolites had the same driving ancestry (Additional File 1: Table S6). For 208 metabolite and local ancestry region associations, the GWAS SNVs did not explain the admixture mapping association (Additional File 3: Extended Data Table S2), suggesting the presence of additional SNVs, likely rare and ancestry-enriched, contributing to the association in these regions.

### Replication of admixture mapping findings

Among 64 metabolites available in the replication sample associated at 169 local ancestry regions, we identified significant associations for 116 of 190 novel metabolite-genomic region associations and 187 of 211 known metabolite-region associations based on a Bonferroni adjusted *p*-value < 1 × 10^−4^ (Additional File 3: Extended Data Table S3). All metabolite-local ancestry associations had the same direction of effects for the ancestry-driving associations.

### Disease relevance

We selected three local ancestry regions for more in-depth analysis based on the large number of metabolites associated and their relevance to disease traits (chromosomes 2, 11, and 15). Two genomic regions (chromosomes 11 and 15) were associated with lipids metabolites driven by Native American ancestry. The chromosome 11 locus included twelve lipid metabolites and seven fine-mapped SNVs that were located at intronic regions of *TMEM258*, *FADS1*, and *FADS2*, and an intergenic SNV that was located near *FTH1* (Additional File 1: Table S5). The chromosome 15 had four associated lipids with a fine-mapped SNV located upstream to *LIPC.* Two lipid metabolites, 1-palmitoyl-2-linoleoyl-GPE (PE) 16:0/18:2 and phosphatidylethanolamine (PE) 18:0/18:2, were associated with local ancestry regions on both chromosomes 11 and 15. *FADS1/FADS2* are involved in the desaturation of fatty acids to generate long-chain polyunsaturated fatty acids (LC-PUFAs). *LIPC* encodes the hepatic triglyceride lipase. Both *FADS1* and *LIPC* code for major enzymes of the LC-PUFA metabolism and have been previously associated with circulating lipids and metabolites [5, 37]. Prior studies have shown evidence for Native American haplotypes at the *FADS1* locus related to reduced PUFA metabolism [38–40], and we additionally identified novel Native American ancestry metabolite associations for SNVs at the *FADS2* and *LIPC*. The ancestral haplogroup of the *FADS* genes, which is associated with a deficient biosynthesis of the biologically active form of PUFA (LC-PUFA), is nearly fixed in Native American populations and has replicated signals of positive selection possibly related to dietary conditions. Within the *FADS* cluster, rs102274, a SNV intronic to *TMEM258* fine-mapped in our analysis (Additional File 1: Table S5), has been considered a causal SNV for LC-PUFA biosynthesis [41]. Signatures of selection in Native American populations for other genes related to diet have also been found in modern populations of Latin America [42].

At the chromosome 2 region, eight N-acetylated amino acids were associated with African local ancestry. Conditional analysis identified seven GWAS SNVs, intronic to *ALMS1/ALMS1P,* that explained the admixture mapping results in the region (Additional File 1: Table S6). Prior studies using blood transcriptome data support associations of N-acetylated amino acid metabolites with *ALMS1* [43]. This gene has been associated to chronic kidney disease [44], a disease with a high burden in individuals of West African descent in the USA.

Additional novel associations of disease relevance are homoarginine at chromosome 15 and carnitine at chromosome 10, for which European ancestry was associated with low circulation levels of these metabolites. Low homoarginine levels are related to endothelial cell dysfunction and cardiovascular disease [45]. The identified association is outside the *GATM* locus at chromosome 15, previously associated with this metabolite in GWAS [46, 47]. Carnitine levels are related to metabolic diseases and mitochondrial function [48].

## Discussion

This study supports the presence of metabolic differences across populations based on ancestry admixture, which are likely driven by genetic ancestral diversity and are potential adaptations to environmental stressors. We identified 2127 significant metabolite-local ancestry associations for 78 metabolites and 651 local ancestry regions within twelve chromosomes. Of the 484 independent local ancestry regions, 232 were novel associations, and several of them were replicated in an independent sample of Hispanics/Latinos. Several associated regions were driven by Native American ancestry, for which genetic variation is less known. Native American is a population less often included in genetic studies. Our study supports the approach of leveraging genetic ancestry to map genes and putative causal variants, as well as to better understand differences in metabolic processes driven by ancestry in admixed populations. The metabolic pathways identified in our study are related to a variety of physiological functions that may be altered in complex diseases or involved in response to environmental stressors, such as those related to diet restrictions and exposure to pathogens. Therefore, our findings have relevance to health and disease.

We identified a wide range of lipid-based metabolites either negatively or positively associated with ancestry-specific genomic regions, predominately on chromosomes 11 and 15, including some newly identified to be associated to these regions. A notable feature of the findings was that many of the metabolites contain LC-PUFAs. For instance, the LC-PUFA arachidonic acid (20:4) of the n-6 biosynthetic pathway (esterified to the glycerol-based backbone of phosphatidylcholines, phosphatidylethanolamines, diacylglycerols, and lysophospholipids) displayed a reduced abundance in individuals with Native American ancestry. A reduction in arachidonic acid in this population could have several biological implications. Arachidonic acid is central in the initiation of inflammatory pathways as it undergoes liberation from phospholipids and serves as a substrate for enzymes, such as cyclooxygenases and lipoxygenases, that generate prostaglandins, leukotrienes, and the newer class of pro-resolution lipoxins [49]. A reduction in these metabolites could suggest an increased production of pro-inflammatory molecules. Some literature suggests differences in inflammatory status in adults and children may be driven by genetic and environmental factors [50]. Future mechanistic studies that tease apart each of these metabolites in primary culture models may shed light on their role in controlling the inflammatory response in the context of specific populations.

Prior literature supports that genetic Native American ancestry haplotypes contribute to a fatty acid desaturase SNV that is associated with low levels of LC-PUFAs of the n-3 biosynthetic pathway [51]. Interestingly, our findings also identified long-chain n-3 PUFAs eicosapentaenoic acid (EPA, 20:5), docosapentaenoic acid, and docosahexaenoic acid (DHA, 22:6) as having a low abundance association with Native American ancestry on chromosome 11. A reduction in EPA and DHA levels is of biological significance as these fatty acids control downstream metabolites (such as resolvins, protectins, and maresins) that drive the resolution of inflammation [52]. Thus, a reduction in these fatty acids may contribute to impaired resolution of inflammation. Finally, it is important to point out that our data revealed changes in PUFA metabolism, notably arachidonic acid, across a wide range of lipid pools, which are likely impacting biological processes outside of inflammation.

In another region, the *ALMS1* locus was associated with an increased abundance of N-acetylated amino acids in African ancestry-derived regions, including some newly associated metabolites. Prior studies have shown evidence of population-specific signals of adaptation in Niger-Congo West Africans at this locus [53]. The mechanisms relating the identified metabolic changes and gene to disease are not fully understood. *ALMS1* has been consistently associated with chronic kidney disease in population studies [44, 54] and in a monogenic disorder (Alstrom syndrome, OMIM #203,800) [33, 55], and obesity and insulin resistance in experimental models [56]. Chronic kidney disease is more common in individuals of African descent in the U.S. Prior studies support African-derived SNVs at another gene (*APOL1*), related to resistance to infectious diseases in Africa, conferring risk to chronic kidney disease in Hispanics/Latinos with West African admixture. A study has shown that ALMS1 protein is involved in the regulation of kidney sodium transport and blood pressure, through interaction with the Na + /K + /2CL-cotransporter (NKCC2) in the nephron loop of Henle [57]. NKCC2 sodium reabsorption is increased in Blacks [58]. These findings provide some mechanistic pathways for the relation among African ancestry at the *ALMS1* locus and chronic kidney disease, but the association with N-acetylated amino acids will require further studies. A nearby gene in this locus, *NAT8*, related to N-acetyltransferase activity, has been associated with N-acetylated amino acids abundance in blood and urine, but gene expression studies showed stronger associations of metabolite-associated SNVs with the *ALMS1* gene [43]. In addition, a study of admixed Brazilians also identified *ALMS1* but not *NAT8* as most significantly associated gene for these metabolites at the region [59].

Several other ancestry-driven genomic regions associated with metabolites were not previously reported, including novel associations for homoarginine and carnitine that have implications for cardiometabolic diseases. These findings support loci discovery and complementary information to GWAS obtained when testing ancestry-driven genomic regions for loci discovery. For some regions previously reported, the GWAS SNV did not explain the admixture mapping associations, supporting the presence of additional causal SNV(s) in these regions. Other approaches that leverage local ancestry for loci discovery and fine-mapping including BMIX [60], which tests association at single markers while stratifying by local ancestry patterns at each interval, could be extended to test three-way ancestry in studies of Hispanics/Latinos and for mixed models. New strategies for fine mapping these regions for potential causal genetic variants are needed that may include integration of sequencing for rare and low-frequency variants and gene expression data generated in admixed populations to better query causal variants that are ancestry enriched. This effort is particularly important to capture Native American ancestry-enriched genetic variants, given the large Native American ancestry proportions in Hispanics/Latinos.

## Conclusions

We identified several ancestry-enriched genomic regions associated with metabolites including Native American-driven regions at chromosomes 11 and 15 related to PUFAs that may contribute to metabolic and inflammatory disease in individuals with Native American ancestry components, and an African-driven genomic region related to N-acetylated amino acid compounds previously identified in chronic kidney disease. These findings support ancestry differences in metabolite regulation of lipid PUFAs and N-acetylated amino acids, which may have implications for common diseases in populations.

## Supplementary Information

**Additional file 1: Table S1.** Descriptive statistics of 3,887 HCHS / SOL participants at visit 1. **Table S2. **Significant metabolites from admixture mapping. **Table S3.** All-ancestries and ancestry-specific admixture mapping results. **Table S4.** Regions whose significance was explained by adding all COJO SNVs to model.

**Additional file 2:** **Fig. S1.** Global ancestry proportions of African, European, and Native American ancestries for participants based on their country of origin: Mainland (Mexico, Central and South America) or Caribbean (Cuba, Dominican Republic, and Puerto Rico). Note that participants from Mainland had a higher proportion of Native American ancestry, while those from Caribbean had a higher proportion of African ancestry. **Fig. S2.** Volcano plots showing relationship between the direction of association and driving ancestry in the three chromosomes with the largest numbers of associated metabolites. The driving ancestry was the ancestry with the smallest p-value in ancestry-specific testing. In chromosome 2, most of the associations with African ancestry were positive. In chromosome 11, most of the associations with Native American ancestry were negative.

**Additional file 3: Extended Data Table S1.** Significant independent local ancestry regions from admixture mapping. **Extended Data Table S2.** Genetic variants that do not explain association between local ancestry regions and metabolites. **Extended Data Table S3. **Replication of significant independent local ancestry regions from admixture mapping for 64 metabolites avaialble in the replication dataset.

## Data Availability

This research was conducted using genotype and phenotype data from HCHS/SOL, which is publicly available through the dbGap (access phs000810.v1.p1). Metabolomics data is available upon request from Dr. Eric Boerwinkle (Eric.Boerwinkle@uth.tmc.edu). The summary results for discovery and replication are included in Additional File 3: Extended Data.

## Abbreviations

eGFR: Estimated glomerular filtration rate
HCHS/SOL: Hispanic Community Health Study/Study of Latinos
HMDB: Human Metabolome Database
GWAS: Genome-wide association study
LC-PUFA: Long-chain polyunsaturated fatty acid
SNV: Single nucleotide variant
