# Supplementary material for: Ancestry-driven metabolite variation provides insights into disease states in admixed populations
Source: Genome Med. 2023 Jul 17;15:52. doi: 10.1186/s13073-023-01209-z (PMC10351197; doi:10.1186/s13073-023-01209-z)
Supplement: Supplementary file 1 — Additional file 1: Table S1. Descriptive statistics of 3,887 HCHS / SOL participants at visit 1. Table S2. Significant metabolites from admixture mapping. Table S3. All-ancestries and ancestry-specific admixture mapping results. Table S4. Regions whose significance was explained by adding all COJO SNVs to model. [file 13073_2023_1209_MOESM1_ESM.pdf]

Additional File 1. Supplementary Tables

Table S1: Descriptive statistics of 3,887 HCHS / SOL participants at visit 1.

**Continuous Variables**	**Mean**	**SD**
Age, yrs	45.9	13.8
eGFR, mL/min/1.73m^2	96.4	19
Global Ancestry*		
European	57	21
African	14	17
Native American	29	23
**Categorical Variables**	**N**	**%**
Sex		
Male	1659	43
Woman	2228	57
Study Center		
Bronx	1025	26
Chicago	875	23
Miami	1104	28
San Diego	883	23
Genetic Group**		
Central American	392	10
Cuban	746	19
Dominican	377	10
Mexican	1405	36
Puerto Rican	705	18
South American	262	7

* Global ancestry is estimated by averaging local ancestry regions across all chromosomes.

** Genetic groups represent patient defined country of origin. eGFR - estimated Glomerular Filtration Rate, a measure of kidney functionality. SD - standard deviation.

Table S2: Significant metabolites from admixture mapping.

Metabolite	RefMet ID	Super Pathway	Mass (m/z)	HMDB ID
adrenate (22:4n6)	Adrenic acid	Lipid	331.2643	HMDB02226
alliin	-	Xenobiotics	178.0532	HMDB33592
3-aminoisobutyrate	-	Nucleotide	104.0706	HMDB03911
2-aminooctanoate	2-Aminooctanoic acid	Lipid	160.1332	HMDB00991
androstenediol (3alpha, 17alpha) monosulfate (3)	-	Lipid	369.1741	-
arachidonate (20:4n6)	Arachidonic acid	Lipid	303.2330	HMDB01043
arachidonoylcholine	-	Lipid	390.3367	-
1-arachidonoyl-GPC (20:4n6)*	-	Lipid	544.3398	HMDB10395
1-arachidonoyl-GPE (20:4n6)*	-	Lipid	502.2928	HMDB11517
1-arachidonoyl-GPI (20:4)*	-	Lipid	619.2889	HMDB61690
1-arachidonylglycerol (20:4)	MG 20:4	Lipid	303.2329	HMDB11549
beta-citrylglutamate	-	Amino Acid	320.0623	-
3beta-hydroxy-5-cholestenoate	-	Lipid	415.3218	-
betaine	-	Amino Acid	118.0863	HMDB00043
butyrylcarnitine (C4)	CAR 4:0	Lipid	232.1543	HMDB02013
carnitine	Carnitine	Lipid	162.1125	HMDB00062
cys-gly, oxidized	-	Amino Acid	355.0741	-
cysteinylglycine	-	Amino Acid	179.0485	HMDB00078
deoxycarnitine	3-Dehydroxycarnitine	Lipid	146.1176	HMDB01161
1,2-dilinoleoyl-GPC (18:2/18:2)	PC 18:2/18:2	Lipid	782.5694	HMDB08138
1,2-dipalmitoyl-GPC (16:0/16:0)	PC 16:0/16:0	Lipid	734.5694	HMDB00564
docosahexaenoate (DHA; 22:6n3)	DHA	Lipid	327.2330	HMDB02183
docosapentaenoate (n3 DPA; 22:5n3)	DPA	Lipid	329.2486	HMDB06528
docosapentaenoate (n6 DPA; 22:5n6)	Docosapentaenoic acid (22n-6)	Lipid	329.2486	HMDB01976
eicosapentaenoate (EPA; 20:5n3)	Eicosapentaenoic acid	Lipid	301.2173	HMDB01999
1-(1-enyl-palmitoyl)-2-arachidonoyl-GPC (P-16:0/20:4)*	PC P-16:0/20:4 or PC O-16:1/20:4	Lipid	766.5745	HMDB11220
1-(1-enyl-palmitoyl)-2-oleoyl-GPC (P-16:0/18:1)*	PC P-16:0/18:1 or PC O-16:1/18:1	Lipid	744.5902	-
1-(1-enyl-palmitoyl)-2-palmitoyl-GPC (P-16:0/16:0)*	PC P-16:0/16:0 or PC O-16:1/16:0	Lipid	718.5745	HMDB11206
1-(1-enyl-palmitoyl)-GPC (P-16:0)*	LPC P-16:0 or LPC O-16:1	Lipid	480.3449	HMDB10407
1-(1-enyl-stearoyl)-2-arachidonoyl-GPE (P-18:0/20:4)*	PE P-18:0/20:4 or PE O-18:1/20:4	Lipid	752.5589	HMDB05779

Table S**2** cont.

**Metabolite**	**RefMet ID**	**Super Pathway**	**Mass (m/z)**	**HMDB ID**
ethylmalonate	-	Amino Acid	131.0350	HMDB00622
homoarginine	-	Amino Acid	189.1346	HMDB00670
5-hydroxylysine	-	Amino Acid	163.1077	HMDB00450
indoleacetylglutamine	-	Amino Acid	304.1292	HMDB13240
1-linoleoyl-2-arachidonoyl-GPC (18:2/20:4n6)*	PC 18:2/20:4	Lipid	806.5694	HMDB08147
1-linoleoyl-2-linolenoyl-GPC (18:2/18:3)*	PC 18:2/18:3	Lipid	780.5538	HMDB08141
linoleoyl-arachidonoyl-glycerol (18:2/20:4) [1]*	DG 18:2$_{2}$0 : 4	Lipid	658.5405	HMDB07257
linoleoyl-arachidonoyl-glycerol (18:2/20:4) [2]*	DG 18:2$_{2}$0 : 4	Lipid	658.5405	HMDB07257
1-linoleoyl-GPC (18:2)	LPC 18:2/0:0	Lipid	520.3398	HMDB10386
1-linoleoyl-GPE (18:2)*	-	Lipid	478.2928	HMDB11507
1-methylimidazoleacetate	-	Amino Acid	141.0659	HMDB02820
N2-acetyllysine	-	Amino Acid	187.1088	HMDB00446
N-acetyl-1-methylhistidine*	-	Amino Acid	212.1028	-
N-acetylarginine	-	Amino Acid	217.1295	HMDB04620
N-acetylasparagine	-	Amino Acid	175.0713	HMDB06028
N-acetylcarnosine	-	Peptide	269.1244	HMDB12881
N-acetylcitrulline	-	Amino Acid	216.0990	HMDB00856
N-acetylglucosaminylasparagine	-	Carbohydrate	336.1402	HMDB00489
N-acetylglutamine	-	Amino Acid	187.0724	HMDB06029
N-acetylkynurenine (2)	-	Amino Acid	251.1026	-
N-acetylleucine	-	Amino Acid	172.0979	HMDB11756
N-acetylphenylalanine	-	Amino Acid	206.0823	HMDB00512
N-acetylputrescine	-	Amino Acid	131.1179	HMDB02064
N-acetyltyrosine	-	Amino Acid	222.0772	HMDB00866
N-delta-acetylornithine	-	Amino Acid	173.0932	-
N-methylpipecolate	-	Xenobiotics	144.1019	-
oleoyl-arachidonoyl-glycerol (18:1/20:4) [1]*	DG 18:1$_{2}$0 : 4	Lipid	660.5562	HMDB07228
2’-O-methyluridine	-	Nucleotide	257.0779	-
6-oxopiperidine-2-carboxylate	-	Amino Acid	142.0510	HMDB61705
5-oxoproline	-	Amino Acid	128.0353	HMDB00267

Supplemental Table 2 cont.

Metabolite	RefMet ID	Super Pathway	Mass (m/z)	HMDB ID
1-palmitoleoyl-2-linolenoyl-GPC (16:1/18:3)*	-	Lipid	754.5381	HMDB08008
1-palmitoyl-2-arachidonoyl-GPC (16:0/20:4n6)	PC 16:0/20:4	Lipid	782.5694	HMDB07982
1-palmitoyl-2-arachidonoyl-GPE (16:0/20:4)*	PE 16:0/20:4	Lipid	740.5225	HMDB05323
1-palmitoyl-2-linoleoyl-GPC (16:0/18:2)	PC 16:0/18:2	Lipid	758.5694	HMDB07973
1-palmitoyl-2-linoleoyl-GPE (16:0/18:2)	PE 16:0/18:2	Lipid	716.5225	HMDB05322
1-palmitoyl-2-stearoyl-GPC (16:0/18:0)	PC 16:0/18:0	Lipid	762.6007	HMDB07970
1-palmitoyl-GPE (16:0)	LPE 16:0/0:0	Lipid	454.2928	HMDB11503
propyl 4-hydroxybenzoate sulfate	-	Xenobiotics	259.0282	-
S-1-pyrroline-5-carboxylate	-	Amino Acid	114.0550	HMDB01301
sphingomyelin (d18:1/20:2, d18:2/20:1, d16:1/22:2)*	-	Lipid	755.6062	-
stearidonate (18:4n3)	Stearidonic acid	Lipid	275.2017	HMDB06547
1-stearoyl-2-arachidonoyl-GPC (18:0/20:4)	PC 18:0/20:4	Lipid	810.6007	HMDB08048
1-stearoyl-2-arachidonoyl-GPE (18:0/20:4)	PE 18:0/20:4	Lipid	768.5538	HMDB09003
1-stearoyl-2-arachidonoyl-GPI (18:0/20:4)	PI 18:0/20:4	Lipid	904.5910	HMDB09815
1-stearoyl-2-linoleoyl-GPC (18:0/18:2)*	PC 18:0/18:2	Lipid	786.6007	HMDB08039
1-stearoyl-2-linoleoyl-GPE (18:0/18:2)*	PE 18:0/18:2	Lipid	744.5538	HMDB08994
1-stearoyl-GPE (18:0)	LPE 18:0/0:0	Lipid	482.3241	HMDB11130
tryptophan betaine	-	Amino Acid	247.1441	HMDB61115

Characteristics of metabolites with at least one significant local ancestry region from admixture mapping. RefMet ID - new lipid naming system devised by the LIPID MAPS consortium.

Table S3: All-ancestries and ancestry-specific admixture mapping results.

		All-Ancestries Analysis		Ancestry-Specific Analysis		
Chr	Metabolites n	Total LA Regions n	Unique LA Regions n	NAM n (%)	AFR n (%)	EUR n (%)
2	13	342	57	-	305 (89.2)	37 (10.8)
4	1	9	9	-	9 (100)	-
5	3	85	85	60 (70.6)	7 (8.2)	18 (21.2)
6	1	2	2	2 (100)	-	-
8	4	52	26	5 (9.6)	26 (50)	21 (40.4)
9	1	6	6	-	6 (100)	-
10	2	95	95	16 (16.8)	-	79 (83.2)
11	38	1206	143	1203 (99.8)	3 (0.2)	-
12	4	62	42	29 (46.8)	33 (53.2)	-
13	1	6	6	-	6 (100)	-
15	8	172	94	118 (68.6)	-	54 (31.4)
16	4	90	86	23 (25.6)	67 (74.4)	-

All significant admixture mapping results. Total local ancestry regions displays the total number of significant regions found on the corresponding chromosome, allowing for region redundancies across metabolites. Unique ancestry regions represents the number of unique local ancestry regions associated with metabolites on the corresponding chromosome. Chr - chromosome, LA - local ancestry; NAM - Native American driving ancestry; AFR - African driving ancestry, EUR - European driving ancestry.

Table S4: Regions whose significance was explained by adding all COJO SNVs to model.

Metabolite	Chr	LA ID	AM Joint P-Value	Conditional Joint P-Value	N COJO SNVs	N GWAS SNVs
N-acetylcitrulline	2	1579	2.05×10^{-31}	1.08×10^{-1}	6	1759
tryptophan betaine	5	4740	1.28×10^{-13}	4.33×10^{-3}	3	5141

When none of the independent COJO SNVs in a local ancestry region explained the association with a metabolite, all of the COJO SNVs for that particular region were included in the admixture mapping model. Chr - chromosome; LA - local ancestry; AM - admixture mapping; N COJO SNVs - number of independent SNVs found within the local ancestry region; N GWAS SNVs - number of SNVs in a region tested in the GWAS analysis.

Table S5: COJO SNVs for local ancestry region 9210 on chromosome 11.

Metabolite	COJO rsID	Gene	Driving Ancestry	Direction of Association	AM Joint P-Value	Conditional Joint P-Value
MG 20:4	rs102274	*TMEM258*	NAM	-	2.15×10^{-37}	$\mathbf{5.72 \times 10^{-3}}$
PC 16:0/18:2	rs11320420	*MYRF*	NAM	+	1.53×10^{-15}	$\mathbf{6.34 \times 10^{-2}}$
Arachidonic acid	rs174533	*MYRF*	NAM	-	1.08×10^{-53}	1.57×10^{-5}
Stearidonic acid	rs174556	*FADS1*	NAM	-	1.30×10^{-19}	$\mathbf{3.60 \times 10^{-4}}$
1-arachidonoyl-GPC (20:4n6)*	rs174562	*FADS2*	NAM	-	7.02×10^{-76}	$\mathbf{1.02 \times 10^{-4}}$
PC 16:0/20:4	rs174562	*FADS2*	NAM	-	1.04×10^{-69}	$\mathbf{1.72 \times 10^{-4}}$
PC 18:0/20:4	rs174562	*FADS1/FADS2*	NAM	-	4.27×10^{-89}	1.94×10^{-5}
arachidonoylcholine	rs174567	*FADS2*	NAM	-	8.82×10^{-28}	$\mathbf{9.13 \times 10^{-3}}$
PC 18:2/18:2	rs3834458	*FADS2*	NAM	+	5.97×10^{-26}	$\mathbf{4.87 \times 10^{-3}}$
LPC 18:2/0:0	rs3834458	*FADS2*	NAM	+	8.16×10^{-11}	$\mathbf{1.23 \times 10^{-1}}$
sphingomyelin (d18:1/20:2, d18:2/20:1, d16:1/22:2)*	rs3834458	*FADS2*	NAM	+	2.24×10^{-10}	$\mathbf{1.61 \times 10^{-2}}$
PE 16:0/18:2	rs5792235	*FADS2*	NAM	+	5.40×10^{-29}	$\mathbf{4.02 \times 10^{-2}}$
PC 18:0/18:2	rs5792235	*FADS2*	NAM	+	3.80×10^{-11}	$\mathbf{1.43 \times 10^{-1}}$
PE 18:0/18:2	rs5792235	*FADS2*	NAM	+	2.32×10^{-22}	$\mathbf{7.67 \times 10^{-2}}$

Local ancestry region 9210 is located between base pairs 61,442,492 and 61,669,946 on chromosome 11. Bold conditional joint p-values are significant ($p > 5 \times 10^{-5}$), meaning the corresponding COJO SNV explains the association between the metabolite and local ancestry region. The direction of association is the sign of the coefficient of the driving ancestry derived from ancestry-specific admixture mapping tests. COJO - conditional and joint association analysis; AM - admixture mapping.

Table S6: COJO SNVs for local ancestry region 1579 on chromosome 2.

Metabolite	COJO rsID	Gene	Driving Ancestry	Direction of Association	AM Joint P-Value	Conditional Joint P-Value
alliin	rs10189885	*ALMS1P1*	EUR	+	2.77×10^{-10}	$\mathbf{9.00 \times 10^{-2}}$
N-acetylkynurenine (2)	rs10189885	*ALMS1P1*	AFR	+	4.13×10^{-12}	$\mathbf{2.87 \times 10^{-1}}$
N-delta-acetylornithine	rs11679202	*ALMS1*	AFR	-	6.15×10^{-24}	9.85×10^{-41}
N2-acetyllysine	rs12611544	*ALMS1P1*	AFR	+	2.87×10^{-17}	4.76×10^{-24}
N-acetylarginine	rs12611544	*ALMS1P1*	AFR	+	4.85×10^{-25}	2.58×10^{-30}
N-acetylcitrulline	rs12611544	*ALMS1P1*	AFR	+	2.05×10^{-31}	1.86×10^{-40}
N2-acetyllysine	rs12620091	*ALMS1P1*	AFR	+	2.87×10^{-17}	$\mathbf{1.19 \times 10^{-3}}$
N-acetylarginine	rs12620091	*ALMS1P1*	AFR	+	4.85×10^{-25}	2.09×10^{-6}
N-acetylcitrulline	rs12620091	*ALMS1P1*	AFR	+	2.05×10^{-31}	1.38×10^{-7}
N-acetylglutamine	rs13431529	*ALMS1P1*	AFR	+	6.05×10^{-12}	$\mathbf{6.29 \times 10^{-1}}$
N-acetylasparagine	rs144594806	*ALMS1*	AFR	+	1.74×10^{-22}	3.46×10^{-29}
N2-acetyllysine	rs1881244	*ALMS1*	AFR	+	2.87×10^{-17}	$\mathbf{3.47 \times 10^{-3}}$
N-acetylarginine	rs1881244	*ALMS1*	AFR	+	4.85×10^{-25}	$\mathbf{3.75 \times 10^{-3}}$
N-acetylasparagine	rs1881244	*ALMS1*	AFR	+	1.74×10^{-22}	$\mathbf{7.78 \times 10^{-4}}$
N-acetylcitrulline	rs1881244	*ALMS1*	AFR	+	2.05×10^{-31}	4.81×10^{-7}
N-acetylglutamine	rs1881244	*ALMS1*	AFR	+	6.05×10^{-12}	$\mathbf{1.42 \times 10^{-2}}$
N-acetylleucine	rs1881244	*ALMS1*	AFR	+	2.07×10^{-19}	$\mathbf{1.52 \times 10^{-4}}$
N-acetyltyrosine	rs1881244	*ALMS1*	AFR	+	1.73×10^{-14}	$\mathbf{1.04 \times 10^{-1}}$
N-acetylarginine	rs188314500	-	AFR	+	4.85×10^{-25}	3.78×10^{-29}
N-delta-acetylornithine	rs200469759	*ALMS1*	AFR	-	6.15×10^{-24}	2.36×10^{-31}
N-acetylasparagine	rs28473697	-	AFR	+	1.74×10^{-22}	4.86×10^{-15}
N-delta-acetylornithine	rs28525015	*ALMS1P1*	AFR	-	6.15×10^{-24}	$\mathbf{3.17 \times 10^{-4}}$
N-acetylleucine	rs28879089	*ALMS1*	AFR	+	2.07×10^{-19}	$\mathbf{6.41 \times 10^{-3}}$
N-acetylasparagine	rs4414703	*NAT8*	AFR	+	1.74×10^{-22}	3.99×10^{-23}
N-acetylkynurenine (2)	rs55745821	-	AFR	+	4.13×10^{-12}	7.70×10^{-10}
N2-acetyllysine	rs57275721	*ALMS1*	AFR	+	2.87×10^{-17}	1.79×10^{-20}
N-acetylarginine	rs57275721	*ALMS1*	AFR	+	4.85×10^{-25}	2.45×10^{-28}
N-acetylcitrulline	rs57275721	*ALMS1*	AFR	+	2.05×10^{-31}	1.41×10^{-35}
N-delta-acetylornithine	rs57275721	*ALMS1*	AFR	-	6.15×10^{-24}	5.00×10^{-27}

Supplemental Table 6 cont.

Metabolite	COJO rsID	Gene	Driving Ancestry	Direction of Association	AM Joint P-Value	Conditional Joint P-Value
N-acetylasparagine	rs61156725	*ALMS1/LOC105374804*	AFR	+	1.74×10^{-22}	9.80×10^{-14}
N-acetylarginine	rs62149659	*LOC105374804*	AFR	+	4.85×10^{-25}	1.57×10^{-50}
N-acetylasparagine	rs62149659	*LOC105374804*	AFR	+	1.74×10^{-22}	1.66×10^{-49}
N-acetylcitrulline	rs62153183	*ALMS1*	AFR	+	2.05×10^{-31}	7.65×10^{-68}
N-acetyltyrosine	rs6546854	*ALMS1*	AFR	+	1.73×10^{-14}	$\mathbf{1.86 \times 10^{-1}}$
N-delta-acetylornithine	rs6715819	*ALMS1*	AFR	-	6.15×10^{-24}	3.67×10^{-13}
N2-acetyllysine	rs7597166	*ALMS1*	AFR	+	2.87×10^{-17}	4.08×10^{-26}
N-acetylarginine	rs7597166	*ALMS1*	AFR	+	4.85×10^{-25}	1.65×10^{-34}
N-acetylcitrulline	rs7597166	*ALMS1*	AFR	+	2.05×10^{-31}	2.62×10^{-41}
N2-acetyllysine	rs79234109	-	AFR	+	2.87×10^{-17}	2.30×10^{-35}

Local ancestry region 1579 is located between base pairs 73,525,537 and 74,051,612 on chromosome 2. Bold conditional joint p-values are significant ($p > 5 \times 10^{-5}$), meaning the corresponding COJO SNV explains the association between the metabolite and local ancestry region. The direction of association is the sign of the coefficient of the driving ancestry derived from ancestry-specific admixture mapping tests. COJO - Conditional and joint association analysis; AM - admixture mapping.

Table S7: COJO SNVs for local ancestry region 11487 on chromosome 15.

Metabolite	COJO rsID	Gene	Driving Ancestry	Direction of Association	AM Joint P-Value	Conditional Joint P-Value
LPE 16:0/0:0	rs1077834	*LIPC*	NAM	+	2.32×10^{-14}	$\mathbf{2.90 \times 10^{-3}}$
LPE 18:0/0:0	rs1077834	*LIPC*	NAM	+	1.85×10^{-11}	$\mathbf{4.26 \times 10^{-2}}$
PE 16:0/20:4	rs2070895	*LIPC*	NAM	+	5.53×10^{-18}	$\mathbf{3.20 \times 10^{-2}}$
PE 18:0/20:4	rs2070895	*LIPC*	NAM	+	5.10×10^{-13}	$\mathbf{1.98 \times 10^{-1}}$

Local ancestry region 11487 is located between base pairs 58,717,919 and 58,742,418 on chromosome 15. Bold conditional joint p-values are significant ($p > 5 \times 10^{-5}$), meaning the corresponding COJO SNV explains the association between the metabolite and local ancestry region. The direction of association is the sign of the coefficient of the driving ancestry derived from ancestry-specific admixture mapping tests. COJO - conditional and joint association analysis; AM - admixture mapping.

Table S8: COJO SNVs for local ancestry region 12348 on chromosome 16.

Metabolite	COJO rsID	Gene	Driving Ancestry	Direction of Association	AM Joint P-Value	Conditional Joint P-Value
cys-gly, oxidized	chr16:89909429	*SPIRE*	NAM	+	1.46×10^{-29}	$\mathbf{4.68 \times 10^{-2}}$
cys-gly, oxidized	rs423135	-	NAM	+	1.46×10^{-29}	2.12×10^{-16}
cysteinylglycine	rs62068366	*SPATA2L*	NAM	+	8.45×10^{-10}	$\mathbf{3.14 \times 10^{-2}}$

Local ancestry region 12348 is located between base pairs 89,726,484 and 90,149,922 on chromosome 15. Bold conditional joint p-values are significant ($p > 5 \times 10^{-5}$), meaning the corresponding COJO SNV explains the association between the metabolite and local ancestry region. The direction of association is the sign of the coefficient of the driving ancestry derived from ancestry-specific admixture mapping tests. COJO - conditional and joint association analysis; AM - admixture mapping.
