# Supplementary material for: Ancestry-driven metabolite variation provides insights into disease states in admixed populations
Source: Genome Med. 2023 Jul 17;15:52. doi: 10.1186/s13073-023-01209-z (PMC10351197; doi:10.1186/s13073-023-01209-z)
Supplement: Supplementary file 2 — Additional file 2: Fig. S1. Global ancestry proportions of African, European, and Native American ancestries for participants based on their country of origin: Mainland (Mexico, Central and South America) or Caribbean (Cuba, Dominican Republic, and Puerto Rico). Note that participants from Mainland had a higher proportion of Native American ancestry, while those from Caribbean had a higher proportion of African ancestry. Fig. S2. Volcano plots showing relationship between the direction of association and driving ancestry in the three chromosomes with the largest numbers of associated metabolites. The driving ancestry was the ancestry with the smallest p-value in ancestry-specific testing. In chromosome 2, most of the associations with African ancestry were positive. In chromosome 11, most of the associations with Native American ancestry were negative. [file 13073_2023_1209_MOESM2_ESM.pdf]

Additional File 2. Supplementary Figures

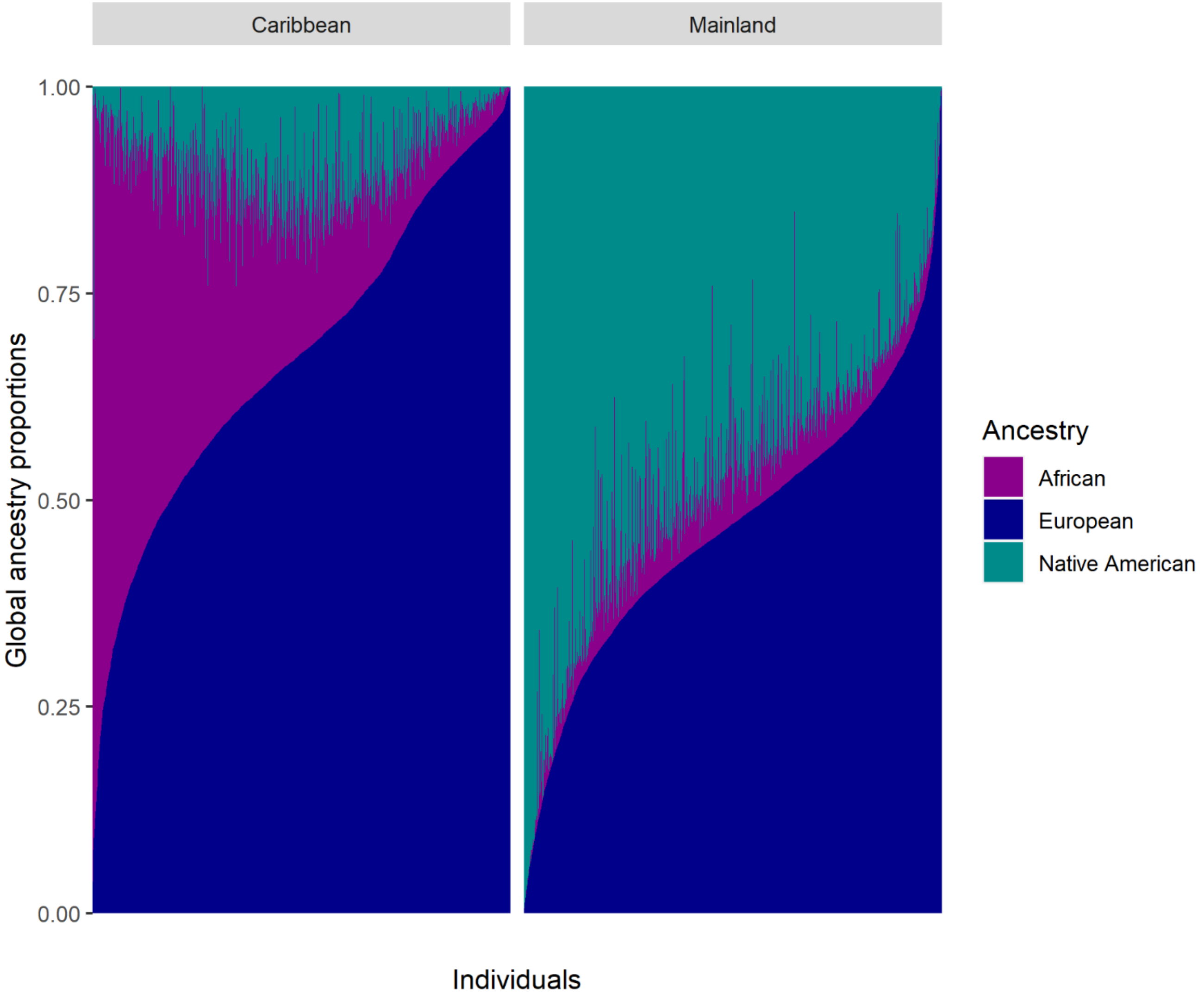

Fig. S1: Global ancestry proportions of African, European, and Native American ancestries for participants based on their country of origin: Mainland (Mexico, Central and South America) or Caribbean (Cuba, Dominican Republic, and Puerto Rico). Note that participants from Mainland had a higher proportion of Native American ancestry, while those from Caribbean had a higher proportion of African ancestry.

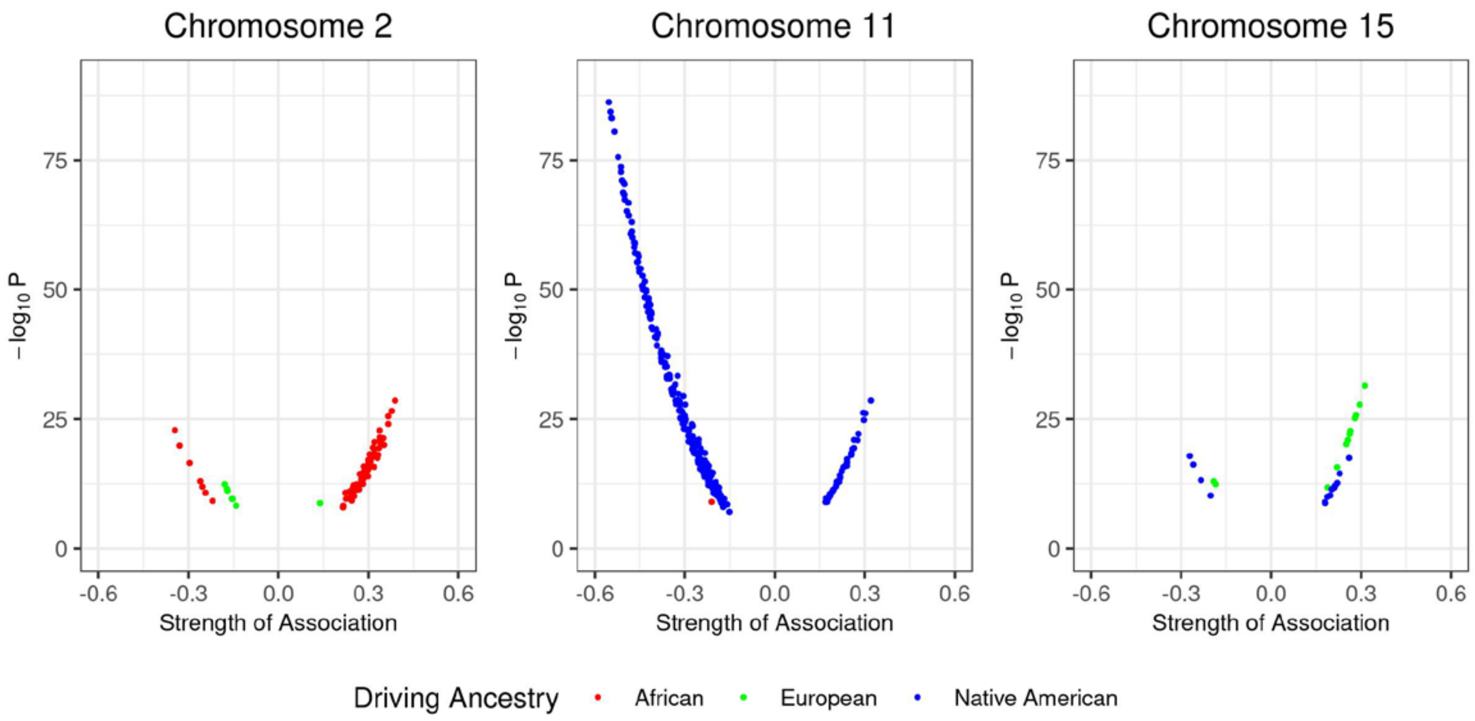

Fig. S2: Volcano plots showing relationship between the direction of association and driving ancestry in the three chromosomes with the largest numbers of associated metabolites. The driving ancestry was the ancestry with the smallest p-value in ancestry-specific testing. In chromosome 2, most of the associations with African ancestry were positive. In chromosome 11, most of the associations with Native American ancestry were negative.
